# A cross‐sectional study on food safety knowledge and practices among food handlers in tertiary and second circle institutions in Ho municipality, Ghana

**DOI:** 10.1002/fsn3.3113

**Published:** 2022-10-26

**Authors:** Felix Kwashie Madilo, Emmanuel Letsyo, Comfort Mawuse Klutse

**Affiliations:** ^1^ Department of Food Science and Technology Ho Technical University Ho Ghana; ^2^ Department of Hospitality and Tourism Management Ho Technical University Ho Ghana

**Keywords:** food handlers, food poisoning, food safety knowledge, food safety practices, foodborne pathogens, Ghana

## Abstract

In recent years, incidences of food poisoning have been reported in some schools across the country. However, little attention has been paid to the hygiene practices of food vendors in the schools. This study, therefore, investigates the food safety knowledge and practices of food vendors catering for tertiary and second cycle students in the Ho municipality. The piloted and validated questionnaire used to sample 608 respondents revealed that the majority of the respondents sampled were female (76.0%), between the ages of 26 and 40 (51%), married (47.4%), and have tertiary or senior high school certificate (60.7%). They have been in business for not more than 2 years (36.2%) and had neither food safety (62.3%) nor good manufacturing practice (81.9%) training. However, they have sufficient knowledge in food safety regarding purchasing, storage, cooking and reheating, and personal hygiene, but exhibited poor knowledge and practice of food temperature control protocols. Both Kendall's tau‐b coefficient correlation and linear regression model revealed a significant positive correlation between food safety knowledge and practices of the vendors. Nevertheless, regular training and monitoring are necessary to enable the vendors to fully implement the food temperature control protocols, which is one of the major causes of food poisoning in the country.

## Graphical abstract

1. The study investigates the food safety knowledge and practices.2. Majority have no food safety and good manufacturing practice training.3. They have sufficient knowledge in food safety with respect to purchasing, storage, and hygiene practices.

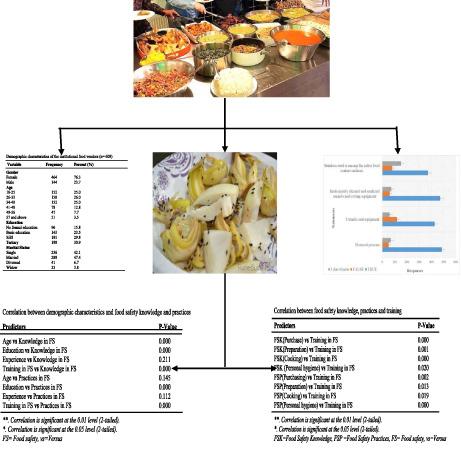

## 1 INTRODUCTION

Food handlers can compromise food safety and hygiene protocols during food preparation, processing, packaging, transportation, storage, and refrigeration. Most often than not, food vendors, due to the location of their vending facilities, prepare food with water from unsafe sources. They have also been found to be engaged in acts, such as inadequate cooking, improper storage and holding time, use of contaminated equipment, improper hand washing, as well as mishandling of refrigeration temperature, that compromise the food safety and personal hygiene standards (Al‐Sakkaf, 2013). Microbial foodborne infections and intoxication occur when food and personal hygiene protocols are breached along the food production chain (Ruby et al., 2019). Food intoxication often begins with diarrheal diseases, then progresses to other serious diseases such as kidney and liver failure, brain and neural disorders, reactive arthritis, cancer, and eventually death (WHO/Yoshi Shimizu, 2022). The inability of the food workers to conform to food safety and hygiene standards during food preparations has been reported in Ghana (Parry‐Hanson et al., 2016), Brazil (Cortese et al., 2016), Saudi Arabia (Ayaz et al., 2018), and South Africa (Mgqibandaba et al., 2020). It is therefore incumbent on the food handlers, most importantly the institutional caterers, to have at least certain skills and knowledge to implement food safety and personal hygiene standards to reduce, if not prevent, the high rate of foodborne infections.

Globally, in 2017, the Center for Disease Control and Prevention (CDC) reported 841 foodborne disease outbreaks, resulting in 14,481 illnesses, 827 hospitalizations, 20 deaths, and 14 food product recalls. The report implicated norovirus as being the most common cause of outbreaks, accounting for 35% of outbreaks and 46% of illnesses. *Salmonella* was the next most common cause, accounting for 29% of outbreaks and 34% of illnesses, followed by Shiga toxin‐producing *Escherichia coli*, which caused 5% of outbreaks and 6% of illnesses, and *Clostridium perfringens*, which caused 5% of outbreaks and 5% illnesses (CDC, 2017). WHO (2017) on the other hand estimated that about 40% of foodborne diseases globally affected children aged 5 years and below.

In Ghana, however, Osei‐Tutu and Anto (2016) and Saba and Gonzalez‐Zorn (2012) revealed that the main foodborne pathogens contaminating foods were *Enterobacter* spp., *Citrobacter* spp., *Klebsiella* spp., and *Escherichia* spp. Yeleliere et al. (2017) also reported *Enterobacter* spp., *Escherichia* spp., *Staphylococcus* spp., and *Pseudomonas* spp. as the most predominant food pathogens identified in Ghanaian foods such as stew, soup, *fufu*, macaroni, salad, and *waakye*. They concluded that the contamination resulted from a lack of food safety knowledge by the food vendors. Der et al. (2009) also reported an outbreak of microbial food poisoning during a child naming ceremony where *Anyaa* Ghana, a locally made drink was served. The study further stated that over 100 girls in a second cycle institution in the Western region were hospitalized after eating contaminated food in the dining hall. Furthermore, Ghana Health Service (2007) reported a number of microbial food poisoning cases among several schools in Madina, Accra, where about 1348 school children suffered food poisoning with several pupils hospitalized.

Several other studies on foodborne disease outbreaks, mainly from improper food handling in Ghana, have been reported over the years. Notably, about 17 of 28 farmers died due to microbial and chemical food poisoning in the Northern region of which a whole family died (Ababio & Lovatt, 2015). Again, Ghana's Food and Drugs Authority (FDA) in 2013 disclosed that about 77% of foodborne disease cases resulted from improper handling among institutional food handlers (FDA, 2013). Furthermore, Osei‐Tutu (2018) revealed improper temperature control and food handling practices, and poor personal hygiene as the main causes of the outbreak of foodborne diseases among the consumers who took part in a buffet lunch served during a conference in Accra, Ghana. On the contrary, Addo‐tham et al. (2020) reported sufficient knowledge of food handlers in the Ejisu‐Juaben municipality of Ghana, while Amegah et al. (2020) found that hand and food hygiene practices among food vendors in educational institutions of the Sagnarigu municipality of Ghana were relatively good (66.0%). However, none of the above studies was conducted among food vendors in the tertiary and second cycle institutions in the country. Students in the universities and senior high schools (SHSs) depend largely on food prepared by these vendors for their survival throughout their stay on the campus, hence, the food they consume must be safe from microbial pathogen contamination.

Elsewhere, however, several authors have investigated the knowledge and practices of food vendors in tertiary institutions; Greek University (Lazou et al., 2012), Lebanese University, Lebanon (Hassan & Dimassi, 2014), universities in Saudi Arabia (Al‐Shabib et al., 2016; Al‐Shabib et al., 2017), public universities in a developing country (Serrem et al., 2021), and Khon Kaen University, Asia (Phyu et al., 2019). These investigations are often used to establish a baseline for the development of effective and relevant food vendors' intervention training programs to reduce, if not prevent, the outbreak of food poisoning in the student population. In Ghana, however, there have been limited studies and information on food safety knowledge and practices among food handlers in and around the environs of universities and SHSs. This study, therefore, investigated the food safety knowledge and practices of tertiary and second cycle food vendors in the Ho municipality of Ghana.

## 2 MATERIALS AND METHODS

### 2.1 Study site description

Ho municipality is one of the 25 administrative districts in the Volta Region of Ghana (Ghana Statistical Service, 2021). The municipality lies between latitudes 6° 207 N and 6° 55 N and longitudes 0° 127 E and 0° 53 E and covers an area of 573.2 km^2^. It shares boundaries with the Adaklu‐Anyigbe District to the South, Hohoe municipal to the North, South‐Dayi district to the West and the Republic of Togo to the East. Ho municipality has a total population of 180,420 constituting about 84,843 (47%) male and 95,577 (53%) female (Ghana Statistical Service, 2021). Ho is the capital city of the municipality as well as the capital of the Volta region, which makes the municipality the largest urban center in the region. The municipality has about 49,826 households with an average size of about four persons per household (Ghana Statistical Service, 2021). The study was carried out in the region due to recent incidents of foodborne disease outbreaks in the region.

The study is a descriptive cross‐sectional survey conducted to examine the food safety knowledge and practices among tertiary and second cycle institutions in the Ho municipality. Fifteen institutions were recruited for the survey. The institutions include four public and private universities, four nursing training colleges, one college of education, and six senior high schools. The research was conducted between May and July 2021.

### 2.2 Sample size and selections

About 608 institutional caterers were conveniently recruited from 15 tertiary and nontertiary institutions in the Ho municipality to participate in the survey using a self‐administered questionnaire. However, a purposive sampling technique was adopted to select the institutions included in the research (i.e., all tertiary and second cycle institutions in the municipality).

### 2.3 Questionnaire development, piloting, and validation

The main instrument used to gather data was a self‐administered questionnaire adapted from Farahat et al. (2015) with some modifications. The questionnaire consisted of four major sections: (1) sociodemographic characteristics which consisted of gender, age, marital status, educational level, and experience; (2) food safety training credentials (three questions); (3) food safety knowledge comprising food purchasing and storage—10 questions, food preparation and temperature control—5 questions, cooking and reheating—8 questions, and personal and kitchen hygiene—12 questions; and (4) food safety practices consisting of purchasing and storage—4 questions, food preparation and temperature control—3 questions, cooking and reheating—5 questions, and personal and kitchen hygiene—8 questions.

The conceptual framework presented the relationship between these variables of the study. Accordingly, the dependent variables for the study were food safety knowledge and practices, while the independent variables included purchase and storage, food preparation and temperature control, cooking and heating, personal and kitchen hygiene, and food safety training.

After the questionnaire was developed, it was reviewed by three food safety experts and the head of the Food Safety Department of FDA, Ghana. The reviewed questionnaire was piloted among five caterers with expertise in food safety. Their feedback was used to modify and validate the content of the questionnaire before rolling it out for data collection. Those involved in the pilot study were eventually excluded from the sampling plan. The scoring technique used by Madilo et al. (2020) was employed to evaluate the respondents. They were scored between 1 and 5, with a higher score representing better food safety knowledge and practices. The rate of the respondents' performance was scaled at 0–69% as inadequate knowledge and practices, and 70%–100% as adequate knowledge and practices. The 5‐point rating scale was preferred over the 7‐point scale as it minimizes the cognitive burden for respondents (i.e., food handlers). The data gathered were statistically analyzed.

### 2.4 Sampling and ethical procedures

The study was finally conducted using a purposive sampling method to select the study areas and a convenient sampling technique to recruit 608 street food business workers for the study. Before the survey was rolled out, permission was sought from the head of the various institutions targeted for the study. Furthermore, ethical approval was sought from the university's research and ethics committee (HTU2022/05/202). Also, all participants approved their participation by providing verbal and written consent. Ethical considerations such as informing participants on the importance of the study, their role, and the overall guiding principle governing the entire study were carried out. The researchers also assured the respondents that any information they would provide would remain confidential and anonymous. Finally, respondents were assured that the findings of this study would not be used for any other purpose but for academic purposes only.

### 2.5 Statistical analysis

The data collected from the completed questionnaires were categorized and presented on excel sheet, imported and coded into the Statistical Package for Social Scientists (SPSS) for windows, version IBM 25.0. Frequencies and percentages of both sociocultural characteristics and the questions testing food safety knowledge and practices were analyzed. Additionally, the questions testing food safety knowledge and practices were transformed and computed separately to obtain mean score for each of the parameters (i.e., knowledge and practices). The mean scores were then used to determine the relationship between food safety knowledge and practices using Kendall's tau‐b nonparametric correlation coefficient, while linear regression model was used to investigate the influence of food safety knowledge on food safety practices.

## 3 RESULT AND DISCUSSION

### 3.1 Demographic characteristics

Table 1 presents the results of the participants' demographic characteristics. In all, about 608 institutional caterers were recruited to participate in the survey. Of this total number, about 76% of the participants were female and 24% were male. The participants who were within the age group of 26 and 40 formed the majority (51%). They were highly educated as majority had tertiary education (30.9%), followed by senior high education (29.8%), and were married (47.4%).

**TABLE 1:** Demographic characteristics of the institutional food vendors (*n* = 608)

Variable	Frequency	%
Gender		
Female	464	76.3
Male	144	23.7
Age		
18–25	152	25.0
26–33	158	26.0
34–40	152	25.0
41–48	78	12.8
49–56	47	7.7
57 and above	21	3.5
Education		
No formal education	96	15.8
Basic education	143	23.5
SHS	181	29.8
Tertiary	188	30.9
Marital status		
Single	256	42.1
Married	288	47.4
Divorced	41	6.7
Widow	23	3.8

### 3.2 Experience, food safety, and good manufacturing practices training of the institutional food venders

The investigators wanted to know how long the caterers have been in the business and whether or not they have any training in food safety and good manufacturing practices (GMP). The results reveal that majority (36.2%) of the caterers were only in the business not more than 2 years. Interestingly, as many as 379 (62.3%) and 498 (81.9%) had neither food safety nor GMP training, respectively. These findings of majority not having food safety or GMP trainings are a cause for concern, particularly as they were catering for students who often experience outbreak of foodborne illnesses on their campuses. This is an indication that the food safety authorities need to step up their efforts to ensure regular training and monitoring of food handlers in and around the schools to protect students from food infection and intoxication. Food safety training should be organized for caterers before the commencement of business, while periodic training at least once a year is highly encouraged to ensure that food prepared for students and the general public is in very high hygienic conditions to avoid foodborne illness cases. A number of similar studies have confirmed the importance of food safety and GMP trainings. Trainings in food safety and GMP are very important instruments for improving knowledge, practice, and attitude of food handlers (Da Cunha et al., 2014). A number of intervention studies conducted through training by Choudhury et al. (2011) and Samapundo et al. (2015) reported that food handlers who were trained had better scores in knowledge, attitude, and practice than their untrained counterparts. Accordingly, training for the institutional catering staff and other food vendors is the only sure way to produce safe food at all times and must be done effectively, continuously, and supervised regularly.

In this study, a self‐reported methodology was employed to determine the food safety knowledge and practices, as well as other factors that may have influenced these practices of food handlers. While there is an inherent weakness (i.e., response bias) in applying this method, steps were taken to strengthen the technique by using anonymity in data collection. Nevertheless, the collection of self‐reported data is believed to be more feasible than observational studies when conducting surveys on behavior or practice in a large population such as the one used in this study.

### 3.3 Food safety knowledge

#### 3.3.1 Food safety knowledge: Purchasing and storage

The findings of food safety knowledge (i.e., in purchasing and storage) are presented in Table 2. The findings indicate that the caterers have sufficient knowledge in food safety in the areas of food purchasing and storage, as majority of them responded correctly to almost all the statements presented to them. More specifically, they were able to exhibit high knowledge in cool storage by indicating that frozen foods should be kept under freezing condition (87.6%), fresh fish must be kept in ice (82.6%), and hot foods must not be kept in chillers (64.8%) with the corresponding mean values of 2.83 ± 0.49, 2.73 ± 0.62, and 2.50 ± 0.77, respectively. These particular responses were expected since almost all food vendors currently use fridges with freezer components. These findings agree favorably with similar studies conducted in Jordan (Osaili et al., 2017) and Saudi Arabia (Alqurashi et al., 2019). Alqurashi et al. (2019), in particular, showed that while about 63.8% of respondents were aware of 4°C as a refrigeration temperature, 72.4% agreed with the statement that the standard temperature for freezers must be −18°C, and also indicated that they must be monitored frequently (77.9%) to avoid spoilage. However, studies by Rebouças et al. (2017) and Stratey et al. (2017) suggested that food handlers exhibited insufficient knowledge in freezing and refrigeration handling.

**TABLE 2:** Food safety knowledge: purchasing and storage (*n* = 608)

Statement	Variable	Response	%	Mean ± SD
1. Should raw food of animal origin be displayed in chillers?				2.48 ± 0.78^a^
	**Yes**	**400**	**65.8**	
	No	101	16.6	
	I do not know	107	17.6	
2. Frozen food should be kept in freezers				2.83 ± 0.49^a^
	**Yes**	**534**	**87.8**	
	No	44	7.3	
	I do not know	30	4.9	
3. Fresh fish should be kept in ice				2.73 ± 0.62^c^
	**Yes**	**502**	**82.6**	
	No	48	7.9	
	I do not know	58	9.5	
4. Grossly unspoiled food can cause food poisoning				2.36 ± 0.79^a^
	**Yes**	**341**	**56.1**	
	No	144	23.7	
	I do not know	123	20.2	
5. First purchased food should be consumed first				2.62 ± 0.71^ac^
	**Yes**	**454**	**74.7**	
	No	75	12.3	
	I do not know	79	13.0	
6. Hot food should not be stored hot in chillers				2.50 ± 0.77^a^
	**Yes**	**394**	**64.8**	
	No	124	20.4	
	I do not know	90	14.8	
7. Opened long life milk should be stored in chillers				2.47 ± 0.76^a^
	**Yes**	**389**	**64.0**	
	No	118	19.4	
	I do not know	101	16.6	
8. Multiplication of food pathogens under optimum condition is 10–30 min				1.77 ± 0.89^b^
	**Yes**	**191**	**31.4**	
	No	85	14.0	
	I do not know	332	54.6	
9. Microbial growth is faster in hot than cold weather				1.92 ± 0.92^a^
	**Yes**	**232**	**38.2**	
	No	94	15.5	
	I do not know	282	46.4	
10. Microbial growth is faster at room temperature than in refrigerators				2.20 ± 0.91^c^
	**Yes**	**323**	**53.1**	
	No	84	13.8	
	I do not know	201	33.1	
11. Microorganisms cannot be destroyed in fridges				2.11 ± 0.89^b^
	**Yes**	**281**	**46.2**	
	No	115	18.9	
	I do not know	212	34.9	
12. Microorganisms cannot be destroyed in freezers				2.15 ± 0.88^a^
	**True**	**357**	**58.7**	
	False	155	25.5	
	I do not know	96	15.8	

*Note*: Correct knowledge are bolded; SD = mean standard deviation; means with same letters as superscripts in a column are significantly different (*p* < .05).

Again, our findings showed, when one sample *t* test was used, that insufficient knowledge in handling food pathogens in storage was significant (*p* < .01) as majority of the respondents do not know or disagree with the statements: “multiplication of food pathogens under optimum condition is 10–30 min” (68.6%), “microbial growth is faster in hot than cold weather” (61.9%), and “microorganisms cannot be destroyed in fridges” (52.8%) with their corresponding mean values of 1.77 ± 0.89, 1.92 ± 0.92, and 2.11 ± 0.89, respectively. This lack of knowledge, however, could have serious health implications, most importantly on the part of the consumers.

#### 3.3.2 Food safety knowledge: Food preparation and temperature control

The results of storage and temperature handling are summarized in Table 3. The findings showed lack or insufficient knowledge of institutional food handlers on how to handle temperature effectively to ensure food safety, with significant difference among the mean values (*p* < .01). It was revealed that only 19.7% understood the fact that prepared salads must not be kept under room temperature. Again, while they showed lack of food safety knowledge by agreeing that frozen foods should be thawed at room temperature (58%), majority did not agree or know that the danger zone of food is between 5 and 63°C (63.8%), with just a little above 50% indicated that raw and cooked foods should be prepared using different cutting boards. Temperature handling during food preparation is very important since food prepared and left at ambient temperature for more than 3 to 4 h enable food pathogens to have access to the food and quickly multiply, which could cause foodborne infections and intoxication when consumed. Parry‐Hanson et al. (2016) and Asiegbu et al. (2016) reported similar findings in their studies. Parry‐Hanson et al. (2016), in particular, stated that majority of their respondents lack knowledge in how freezing and defrosting of food are properly done. They quickly added that it would be dangerous to the consumers if this temperature mismanagement is not corrected.

**TABLE 3:** Food safety knowledge: food preparation and temperature control (*n* = 608)

Statement	Variable	Response	%	Mean ± SD
13. Prepared salad should be kept at room temperature for the entire catering period				0.43 ± 0.75^b^
	Yes	291	47.9	
	**No**	**120**	**19.7**	
	I do not know	197	32.4	
14. Frozen food should be thawed at room temperatures				2.39 ± 0.79^c^
	True	355	58.4	
	**False**	**134**	**22.0**	
	I do not know	119	19.6	
15. Frozen food can be completely thawed and refrozen				2.41 ± 0.77^b^
	**True**	**351**	**57.7**	
	False	155	25.5	
	I do not know	102	16.8	
16. Raw and cooked food can be prepared using the same cutting boards				2.25 ± 0.66^b^
	True	228	37.5	
	**False**	**307**	**50.5**	
	I do not know	73	12.0	
17. The danger zone of food is between 5–63°C				1.84 ± 0.93^b^
	**True**	**220**	**36.2**	
	False	71	11.7	
	I do not know	317	52.1	

*Note*: Correct knowledge is bolded; SD = mean standard deviation; Means with same letters as superscripts in a column are significantly different (*p* < .05).

#### 3.3.3 Food safety knowledge: Cooking and reheating

The respondents' food safety knowledge in cooking and reheating was tested and the findings are presented in Table 4. The food vendors were able to show sufficient knowledge in food safety during cooking and reheating. Most importantly, majority of the caterers indicated that prepared food should not be kept for more than 4 h outside the chillers (55.3%), half cooked food of animal origin can lead to food poisoning (71.7%), and inadequately reheated cooked food can cause food poisoning (71.5%) with their corresponding means as 2.28 ± 0.87, 2.54 ± 0.78, and 2.57 ± 0.73, respectively. These findings agree favorably with the studies of Lestantyo et al. (2017) in Semarang, Central Java and Lee et al. (2017) in Kuala Lumpur which indicated that food handlers or caterers have sufficient knowledge in food handling procedures and food poisoning issues. However, respondents in this study failed to acknowledge that thermometer should always be used to cook raw meat (34.2%). This is an indication that these institutional caterers might have been either preparing food which is not properly cooked or overcooked. The mean values presented differ significantly (*p* < .01).

**TABLE 4:** Food safety knowledge of the respondents in cooking and reheating

Statement	Variable	Response	%	Mean ± SD
19. Prepared food should not be kept for more than 4 h outside the chillers				2.28 ± 0.87^a^
	**Yes**	**336**	**55.3**	
	No	106	17.4	
	I do not know	166	27.3	
20. Half cooked food of animal origin can lead to food poison				2.54 ± 0.78^a^
	**Yes**	**436**	**71.7**	
	No	64	10.5	
	I do not know	108	17.8	
21. Inadequately reheated cooked food can cause food poison				2.57 ± 0.73^a^
	**Yes**	**435**	**71.5**	
	No	86	14.1	
	I do not know	87	14.3	
23. Food must not be reheated more than once				2.29 ± 0.81^a^
	**Yes**	**312**	**51.3**	
	No	159	26.2	
	I do not know	137	22.5	
24. Thermometer should always be used to cook raw meat in particular				1.93 ± 0.87^b^
	**Yes**	**208**	**34.2**	
	No	151	24.8	
	I do not know	249	41.0	

*Note*: Correct knowledge are bolded; SD = mean standard deviation; means with same letters as superscripts in a column are significantly different (*p* < .05).

#### 3.3.4 Food safety knowledge: Personal and kitchen hygiene

**FIGURE 1:**
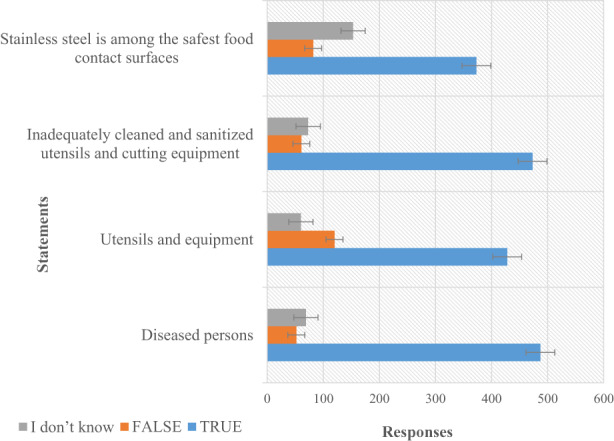
Knowledge of the sources of microbial food contamination

Table 5 represents the results of the respondents' personal and kitchen hygiene knowledge. With a careful analysis of the results, it was discovered that the institutional caterers exhibited sufficient knowledge in personal hygiene as more than 80% of the respondents agreed to all the positive statements concerning personal hygiene. Specifically, they agreed that ill persons with gastroenteritis (stomach flu) should not handle food (82.4%), foods must not be tasted with hands during cooking (81.7%), and hands should be properly cleaned during food preparation (90.3%) with the mean values of 2.74 ± 0.59, 2.77 ± 0.51, and 2.86 ± 0.46, respectively (*p* < .01). Previous studies, Osaili et al. (2017) and Bou‐Mitri et al. (2018), have reported sufficient knowledge of food handlers on personal hygiene and cross‐contamination. In contrast, Table 5 suggests that the caterers showed inadequate knowledge in kitchen hygiene. They demonstrated that tap water with detergent (51.8%) should be used instead of warm water with detergents (33.6%) and using special towels for drying food utensils and equipment (43.8%) instead of disposable tissues (30.4%). This suggests that the respondents have insufficient knowledge in cross‐contamination which needs to be addressed with immediate effect to avert foodborne outbreaks among the student communities. Furthermore, the researchers wanted to find out if the caterers have any knowledge in some of the sources of contamination by food pathogens. From Figure 1, it was very clear that they have sufficient knowledge as they agreed to the fact that pathogens in food could have come from diseased person (80.0%), utensils and equipment (70.1%), and inadequate sterilization of utensils and cutting boards (77.8%).

**TABLE 5:** Food safety knowledge in relation to personal and kitchen hygiene (*n* = 608)

Statement	Variable	Response	%	Mean ± SD
27. Food handling should be avoided with symptoms of gastroenteritis (stomach flu)				2.74 ± 0.59^c^
	**True**	**501**	**82.4**	
	False	58	9.5	
	I do not know	49	8.1	
28. Cooked food should not be tasted by fingers or unclean spoons				2.77 ± 0.51^c^
	**True**	**497**	**81.7**	
	False	84	13.8	
	I do not know	27	4.4	
To prepare safe food, hands should be:				
29. Properly cleaned				2.86 ± 0.46^a^
	**True**	**549**	**90.3**	
	False	32	5.3	
	I do not know	27	4.4	
30. Free of wounds				2.83 ± 0.49^a^
	**True**	**535**	**88.0**	
	False	44	7.2	
	I do not know	29	4.8	
31. With short and clean nails				2.81 ± 0.53^b^
	**True**	**533**	**87.7**	
	False	35	5.8	
	I do not know	40	6.6	
67. Cleaning of food utensils and equipment should be done using:				2.19 ± 0.67^c^
	Tap water	89	14.6	
	Tap water and detergent	315	51.8	
	**Warm water and detergent**	**204**	**33.6**	
68. Sterilizing food utensils and equipment should be done using:				1.63 ± 0.73^c^
	**Chlorine**	**89**	**14.6**	
	Steam	203	33.4	
	Others		316	52.0
70. Drying food utensils and equipment must be done by:				2.05 ± 0.75^b^
	Inverting them	157	25.8	
	Special towel	266	43.8	
	**Disposal tissue**	**185**	**30.4**	

*Note*: Correct knowledge are bolded; SD = mean standard deviation; means with same letters as superscripts in a column are significantly different (*p* < .05).

### 3.4 Food safety practices

In terms of food safety practices in food purchasing and storage, the respondents were able to exhibit beyond reasonable doubt that they put the knowledge they have in food safety into practice (Table 6). Most importantly, they indicated that they often read expiring dates before purchasing (56.4%) and also often avoid storing cooked foods while still hot in the chiller (51.2%). When the caterers were asked if they avoid storing cooked and left‐over foods in the chillers for more than 3 days, majority (49.8%) indicated that they have always done it (Table 6). Even though the respondents exhibited sufficient food safety practices regarding food preparation and storage, it was however observed (Table 7) that standards regarding temperature control were often compromised. Majority of the caterers washed their salad vegetables under running water (38.7%). They used other means of thawing frozen foods of animal origin rather than in chiller (21.2%) and failed to keep stews and soup above 73°C (39.5%). Significant differences (*p* < .01) existed among the figures presented. Compromising food safety standards is the surest cause of food contamination and foodborne illness outbreaks, and training in food safety and GMP coupled with awareness creation could easily help to avoid this unfortunate incidence.

**TABLE 6:** Food safety practice: purchasing and storage (*n* = 608)

Statement	Variable	Response	%	Mean ± SD
38. Reading expiry date before purchasing				4.28 ± 1.00^a^
	**Often**	**343**	**56.4**	
	Sometimes	145	23.8	
	Never	20	3.3	
	Seldom	83	13.7	
	Indifferent	17	2.8	
39. Purchasing food of animal origin displayed refrigerated				4.10 ± 0.99^b^
	**Often**	**252**	**41.4**	
	Sometimes	230	37.8	
	Never	29	4.8	
	Seldom	79	13.0	
	Indifferent	18	3.0	
41. Avoiding storage of cooked food while still hot in chiller				4.11 ± 1.16^b^
	**Often**	**311**	**51.2**	
	Sometimes	164	27.0	
	Never	57	9.3	
	Seldom	50	8.2	
	Indifferent	26	4.3	
51. Avoiding storing of cooked and leftover foods in the chillers for more than 3 days				
	**Often**	**303**	**49.8**	
	Sometimes	203	33.4	
	Never	102	16.8	
	Seldom	0	0	
	Indifferent	0	0	2.34 ± 0.76^b^

*Note*: Correct practices are bolded; SD = mean standard deviation; means with same letters as superscripts in a column are significantly different (*p* < .05).

**TABLE 7:** Food safety practice: food preparation and temperature control (*n* = 608)

Statement	Variable	Response	%	Mean ± SD
42. Washing of salad vegetables:				2.63 ± 1.22^a^
	Soaking in water	119	19.6	
	**Under running water**	**235**	**38.7**	
	Soaking in water with vinegar	27	4.4	
	Soaking in water with salt	227	37.3	
43. Thawing frozen food of animal origin is usually done:				3.26 ± 1.26^b^
	During cooking of small pieces	42	6.9	
	By soaking in water	170	28.0	
	Under running water	110	18.1	
	Over the kitchen counter	157	25.8	
	**In the chiller**	**129**	**21.2**	
26. Stews and soups should be kept above 73°C until served				1.88 ± .96^b^
	**Yes**	**240**	**39.5**	
	No	51	8.4	
	I do not know	316	52.0	

*Note*: Correct practices are bolded; SD = mean standard deviation; means with same letters as superscripts in a column are significantly different (*p* < .05).

Moreover, researchers wanted to know if the respondents have sufficient practice in food safety regarding cooking and reheating. The results of this assessment are illustrated in Table 8 with significant differences among the means (*p* < .01). Table 8 reveals that majority of the food handlers do not leave cooked food in the kitchen till eaten for more than 4 h (43.3%), very often heat food adequately before eating (68.2%), and often reheat food portion sufficiently for a meal (62.8%). However, they could not prove that they never cook food in quantity sufficient for less than 3 days (12.5%) and use thermometer to check adequacy of food during cooking (7.4%). In this case, it was observed that their food preparation knowledge (Table 3) has significantly reflected in practices.

**TABLE 8:** Food safety practice: cooking and reheating (*n* = 608)

Statement	Variable	Response	%	Mean ± SD
49. Not leaving cooked food in the kitchen till eating for more than 4 h				2.26 ± 0.79^c^
	**Often**	**263**	**43.2**	
	Sometimes	229	37.7	
	Never	116	19.1	
	Seldom	0	0.0	
	Indifference	0	0.0	
50. Cooking of food in quantities sufficient for less than 3 days				2.44 ± 0.71^c^
	Often	324	53.3	
	Sometimes	208	34.2	
	**Never**	**76**	**12.5**	
	Seldom	0	0.0	
	Indifference	0	0.0	
53. Adequate reheating of foods				2.61 ± 0.62^a^
	**Often**	**415**	**68.2**	
	Sometimes	149	24.5	
	Never	44	7.2	
	Seldom	0	0.0	
	Indifference	0	0.0	
54. Reheating of a potion sufficient for a meal				2.53 ± 0.71^b^
	**Often**	**382**	**62.8**	
	Sometimes	160	26.3	
	Never	66	10.9	
	Seldom	0	0.0	
	Indifference	0	0.0	
55. Checking adequacy of food cooking by:				2.10 ± 0.84^a^
	Examining the texture by a fork	130	21.4	
	Tasting	325	53.4	
	Examining internal and external color change	108	17.8	
	**Under thermometer**	**45**	**7.4**	

*Note*: Correct practices are bolded; ± = mean standard deviation; means with same letters as superscripts in a column are significantly different (*p* < .05).

#### 3.4.1 Food safety practices: Personal and kitchen hygiene

The results of food safety practices in relation to personal and kitchen hygiene are presented in Table 9. It was interesting to note that the high level of food safety knowledge regarding personal hygiene exhibited by the vendors reflected in their practices. Majority of the respondents demonstrated that they often wash hands before beginning food preparation (81.95%), often wash hands after visiting washroom (83.7%), and wash, sterilize, and dry food utensils and equipment very often (68.1%). Additionally, they also revealed that they do not cook when they are ill (65.8%) and always wash hands using water and soap (62.2%). The mean values differ significantly. This was a clear indication that even though the food handlers might not have standard food safety and hygiene training or workshops, they might be practicing food safety protocols due to their previous knowledge from home or might have observed the protocols from others. Proper personal, kitchen, and food hygiene practices should be employed to drastically reduce foodborne disease outbreaks such as diarrhea and gastrointestinal foodborne infections and intoxications.

**TABLE 9:** Respondents' food safety practices regarding personal and kitchen hygiene (*n* = 608)

Statement	Variable	Response	%	Mean ± SD
56. I avoid food preparation when ill				2.60 ± 0.63^a^
	**Often**	**400**	**65.8**	
	Sometimes	166	27.3	
	Never	42	6.9	
	Seldom	0	0.0	
	Indifference	0	0.0	
57. I wash hands using warm water and soap				2.54 ± 0.66^a^
	**Often**	**378**	**62.2**	
	Sometimes	176	28.9	
	Never	54	8.9	
	Seldom	0	0.0	
	Indifference	0	0.0	
59. I wash hands before food preparation				2.79 ± 0.48^a^
	**Often**	**498**	**81.9**	
	Sometimes	90	14.8	
	Never	20	3.3	
	Seldom	0	0.0	
	Indifference	0	0.0	
60. I wash hands after using the WC				2.79 ± 0.50^b^
	**Often**	**509**	**83.7**	
	Sometimes	73	12.0	
	Never	26	4.3	
61. I avoid tasting of cooked food by fingers				2.55 ± 0.68^c^
	**Often**	**399**	**65.6**	
	Sometimes	143	23.5	
	Never	66	10.9	
	Seldom	0	0.0	
	Indifference	0	0.0	
62. I avoid tasting of cooked food by inserting the same spoon several times				2.46 ± 0.69^a^
	**Often**	**344**	**56.6**	
	Sometimes	201	33.1	
	Never	63	10.3	
	Seldom	0	0.0	
	Indifference	0	0.0	
64. I sterilize food utensils and equipment				2.46 ± 0.69^a^
	**Often**	**341**	**56.1**	
	Sometimes	204	33.6	
	Never	63	10.3	
	Seldom	0	0.0	
	Indifference	0	0.0	
65. I dry food utensils and equipment				2.62 ± 0.598^b^
	**Often**	**414**	**68.1**	
	Sometimes	157	25.8	
	Never	37	6.1	
	Seldom	0	0.0	
	Indifference	0	0.0	

*Note*: Correct practices are bolded; ± = mean standard deviation; means with same letters as superscripts in a column are significantly different (*p* < .05).

Comparing the results of this study to previous similar studies, our results correlated poorly with reports from New York (Burt et al., 2016), Spain (Garayoa et al., 2011), and Brazil (Cortese et al., 2016), where less than 20% of the food handlers were observed conforming to the personal hygiene standards. Notably, in Spain, catering companies have observed missing cap (85%), unavailable masks (50%), and presence of jewelries in food items (60%) (Garayoa et al., 2011). However, a positive correlation was observed with a study by Sani and Siow (2014), where about 98.2% of the respondents washed hands before and after touching foods and their body parts and 82.8% approved the statement, “food handlers with abrasion or cuts on fingers and hands and/or sick must not handle foods.” A similar positive correlation was reported when about 92% of the food handlers indicated that they know touching and tasting food with cut hands and fingers is dangerous (Tokuc et al., 2009).

### 3.5 Correlation between food safety knowledge and food safety practices

Kendall's tau‐b coefficient was used to investigate the bivariate correlation between food safety practices and knowledge of the food handlers with respect to purchasing, preparation, and cooking. The results reported in Table 10 reveal that there was a significant positive correlation (*p* < .01) between food safety knowledge and practices of the institutional caterers. This implies that sufficient knowledge of the food handlers on food safety issues will definitely impact their food safety practices. However, similar investigations conducted by Abdul‐Mutalib et al. (2012), Sani and Siow (2014), Rosnani et al. (2014), and Alqurashi et al. (2019) reported significant differences between food safety knowledge and practices.

**TABLE 10:** Correlation between food safety knowledge and practices

Predictors	Correlation coefficient	*p* Value
FSK (purchase) versus FSP (purchasing)	.178**	.000
FSK (preparation) versus FSP (preparation)	.082**	.008
FSK (cooking) versus FSP (cooking)	.205**	.000

Abbreviations: FSK, food safety knowledge; FSP, food safety practices.

** Correlation is significant at the .01 level (two‐tailed).

Table 11 also presents the impact an effective food safety training would have on food safety knowledge and practices. The findings established that food safety training has a significant relationship (*p* < .01; *p* < .05) with food safety knowledge and practices with respect to food purchasing, preparation, and cooking. Food vendors' training programs are supposed to influence safe food handling behavior in their vending workplace. However, these knowledge‐based training programs do not always correspond to safe food handling (Clayton & Griffith, 2008). There is therefore a need to use behavioral science theories to understand the correlation between food safety training and the knowledge and practices of vendors. The main theoretical framework for this study was Bandura's Social Cognitive Theory (SCT), which is often used to explain how humans acquire, maintain, and apply certain behaviors (Bandura, 2012). The findings of the study support the SCT in that food purchasing, preparation, and cooking knowledge and practices improved with food safety training. To improve the retention of knowledge and practices, food safety training programs based on the SCT should be more interactive and the use of incentives such as certification and special awards should be encouraged.

**TABLE 11:** Correlation between food safety knowledge, practices, and training

Predictors	Correlation coefficient	*p* Value
FSK (purchase) versus training in FS	.226**	.000
FSK (preparation) versus training in FS	.133**	.001
FSK (cooking) versus training in FS	.213**	.000
FSK (personal hygiene) versus training in FS	.094*	.020
FSP (purchasing) versus training in FS	.126**	.002
FSP (preparation) versus training in FS	.101*	.013
FSP (cooking) versus training in FS	.095*	.019
FSP (personal hygiene) versus training in FS	.162**	.000

Abbreviations: FSK, food safety knowledge; FS, food safety; FSP, food safety practices.

** Correlation is significant at the .01 level (two‐tailed);

* Correlation is significant at the .05 level (two‐tailed).

With regards to the relationship that might exist between the demographic characteristics of the institutional caterers and their food safety knowledge and practices, Table 12 demonstrates that education and training have impacted both food safety knowledge and practices (*p* < .01). The findings also revealed that even though positive relationship has been established with food safety knowledge (*p* < .01), it failed to do same with food safety practices (*p* ˃ .05). Surprisingly, experience of the caterers had no relationship with neither food safety knowledge nor practice (Table 12).

**TABLE 12:** Correlation between demographic characteristics and food safety knowledge and practices

Predictors	Correlation coefficient	*p* value
Age versus knowledge in FS	−.172**	.000
Education versus knowledge in FS	.346**	.000
Experience versus knowledge in FS	−.051	.211
Training in FS versus knowledge in FS	.185**	.000
Age versus practices in FS	−.044	.145
Education versus practices in FS	−.237**	.000
Experience versus practices in FS	−.050	.112
Training in FS versus practices in FS	.167**	.000

Abbreviations: FS, food safety.

** Correlation is significant at the .01 level (two‐tailed).

* Correlation is significant at the .05 level (two‐tailed).

### 3.6 Influence of food safety knowledge on food safety practices using linear regression model

In this study, we hypothesized that there is a positive correlation between food safety knowledge and practices of food vendors in both the tertiary and second circle institutions. Accordingly, a linear regression model was designed to investigate whether or not food safety knowledge has any influence on the respondents' food safety practices. The results summarized in Table 13 indicated that food safety knowledge of the institutional caterers has a significant positive influence on the food safety practices of the respondents, which was also corroborated by Kendall's tau‐b coefficient correlation. The model revealed that food safety knowledge can predict practices statistically (*F* = 191.29; *df* = 1; *p = .01). About 0.02% variability in safety practices was accounted for by food safety knowledge. This implies that respondents with a mean value 13.83 of food safety knowledge would be expected to averagely have a safety practice of 33.575. Alqurashi et al. (2019) and Abdul‐Mutalib et al. (2012) reported similar findings in similar investigations.

**TABLE 13:** Influence of food safety knowledge on food safety practice using a linear regression model

	Mean	Std. dev	Constant	*B*	*p* Value	Adjusted *R* ^2^
Food safety practice (dependent variable)	17.87	1.88	33.575	–	–	.239
Food safety knowledge (independent variable)	13.83	0.02	–	0.307	.000*	

*Note*: *F* = 191.29, *df* = 1.

* *p* < .01, unstandardized coefficient *B* = 0.307.

### 3.7 Influence of demographic characteristics on food safety knowledge and practices using linear regression model

Again, the investigators wanted to find out if the demographic characteristics of the food handlers would have any influence on their food safety knowledge and practices using linear regression models. Table 14 presents the summary of the outcome of the investigation. The models were significant (*p* < .01), with the independent variables accounting for 24.5% of safety knowledge (*R* ^2^ = .245) and 34.1% of safety practices (*R* ^2^ = .341). This shows a strong positive relationship between food safety knowledge and practices and a positive correlation between education and knowledge and practice and between food safety training and knowledge and practices. These results were consistent with Alqurashi et al. (2019) who reported positive correlation between educational level and food safety knowledge and practices. However, our findings (Table 14) also indicate that age, gender, and experience have no influence on food safety knowledge and practices of the food handlers.

**TABLE 14:** Influence of demographic characteristics on food safety knowledge and food safety practices using a linear regression model

Independent variables	Dependent variable (coefficient)	
	Food safety knowledge	Food safety practices
Age	0.064	0.997
Gender	0.057	0.415
Education	0.000	0.000
Experience	0.743	0.098
Training in food safety	0.000	0.000

** *p* < .01.

## 4 CONCLUSION

Institutional caterers play a crucial role in and around schools and universities as students depend largely on the foods they prepare for their survival throughout their stay on the campuses. However, in recent years, there has been an outbreak of foodborne illness among students who consumed contaminated food from these caterers. In view of this, we conducted a study to assess the food safety knowledge and practices among institutional caterers in the Ho municipality of Ghana. The study revealed that the caterers are relatively young (between 18 and 33 years) and were mostly in the business for not more than 2 years. It also emerged that most of the caterers have neither food safety nor GMP training, as well as inadequate food safety knowledge in kitchen hygiene and temperature control protocols. Nevertheless, most of them exhibited high food safety knowledge in handling food during preparations, personal hygiene, and cool and hot storage protocols. With regard to food safety practices, most caterers observed the food safety protocols with respect to food preparation and storage; however, most of them compromise on the standards regarding temperature control. In line with these findings, food safety and GMP training interventions are imperative to update the knowledge of the caterers and significantly improve their current practices, most importantly the knowledge and practices of food temperature control where they exhibited so much deficiency. Moreover, the study could be used to establish a baseline for the development of effective and relevant food vendors' intervention training programs. It is recommended that regular monitoring should be conducted across the study area to prevent food poisoning incidences. Further studies should be carried out to assess students' knowledge or awareness of foodborne pathogens and their sources. While there was always the possibility of guessing on a self‐administered questionnaire, the presence of an “I do not know” option on the instrument and anonymity during data collection should have improved the internal validity of the study thereby reducing the possibility of interviewer bias.

## FUNDING INFORMATION

This research did not receive any specific grant from funding agencies in the public, commercial, or not‐for‐profit sectors.

## CONFLICT OF INTEREST

The authors have declared no conflicts of interest for this article.

## Data Availability

The data that support the findings of this research are available in the manuscript.
